# Checklist of the family Epitoniidae (Mollusca: Gastropoda) in Taiwan with description of a new species and some new records

**DOI:** 10.3897/BDJ.4.e5653

**Published:** 2016-07-19

**Authors:** Chih-Wei Huang, Yen-Chen Lee

**Affiliations:** ‡ National Taiwan Normal University, Taipei City, Taiwan; § Biodiversity Research Center, Academia Sinica, Taipei, Taiwan

**Keywords:** Epitoniidae, new records, fauna, East Asian, Indo-Pacific region, Taiwan

## Abstract

**Background:**

The family Epitoniidae is a group of small to medium-sized gastropods and occurs globally from the intertidal zone to abyssal seabeds. There are 101 epitoniid species currently recorded from Taiwan.

**New information:**

Based on our investigations of seashores and fishing ports of Taiwan, a new species and 12 new records of Epitoniidae species are reported. Of the 12 new records, four are new to the East Asian region and two are new records to the Indo-Pacific region. Our results increase the number of Taiwanese Epitoniidae from 101 species to 114 species.

## Introduction

The family Epitoniidae is a group of medium to small size gastropods, usually associating with cnidarians (Gittenberger et al. 2000) and found globally from the intertidal zone to abyssal seabeds. However, most epitoniid species are rare, only a few individuals are provided to study, and most are represented only in private collections (Nakayama 2003). Early naturalists such as Sowerby (1844a), Sowerby (1844b), Reeve (1849), Tryon (1887), Clessin (1897), de Boury (1889), Jousseaume (1911) and Jousseaume (1911) reported many new species and described the taxonomy of this family worldwide. In recent decades, a few local fauna have been reported for the Atlantic species (Bouchet and Waren 1986, DuShane 1974), South African species (Kilburn 1985), and Japanese species (Nakayama 2003).

Kilburn (1985), in his work on the South African Epitoniidae fauna, stated that ‘epitoniid taxonomy remains in a chaotic state, particularly above the species level’. There had been no systematic discussion of the family Epitoniidae in the Taiwan waters and the adjacent area since Kuroda (1941) reported 12 species of this family, including four unidentified species.

Since Kuroda, Hayasaka (1944) reported a new living record Taiwanese species and two fossil species. Lan (1976) and Lan 1999reported a new species (currently recognized as synonym) and one new record from Taiwan. Shih and Wan (1982) illustrated five species, although two were misidentified. Actually, they reported three species, including one new record from Taiwan. Chong (1993) illustrated a new record of a Taiwanese species, and Lai (1998) illustrated the other two additional new records from Taiwan. Su (1999) illustrated nine species including four new records from Taiwan. Lee and Wu (1998) and Lee (2001b) reported an additional six new species, and Lee (2001a) and Lee (2003) illustrated seven new records from Taiwan. Chen and Lee (2007) illustrated nine tiny epitoniids, six of which were new records from Taiwan. From our viewpoint, most of the earlier report epitoniids are misidentified. A recent published work of Taiwan epitoniids (Lee and Wu 2012) solved the scientific name chaos and reported a huge amount of new records epitoniids from Taiwan. However, new species and records are being discovered continually.

In this study, we will report a new species and 12 new records of epitoniids from Taiwan.

## Materials and methods

The epitoniids in this study were collected directly from seashores and fishing ports in Taiwan. The specimens were taken back to the laboratory and cleaned for identification. The habitat depth of the epitoniids are based on the fishing grounds of some particular prawns (Yu and Chan 1986) and lobsters (Chan and Yu 1993), which were trawled with the epitoniids. The distributions of epitoniids are based a shrimp boat logbook. The specimens of holotype and paratype of Epitonium (Parviscala) corniculum sp. n. are deposited in the National Museum of Natural Science, Taiwan (NMNS-7035-001~003).

## Taxon treatments

### Claviscala subulae

Nakayama 2000

#### Description

Dull white, elongated, spiral elevated with 12 whorls (Fig. 1a). Surface with about 16 weak thick and low axial ribs on the body whorl, spiral striae strong, crossed over axial ribs, about 9 in number on the body whorl, with a basal disc. Shell height 11–15mm.

#### Distribution

This species occurs off Choshi, Boso Peninsula, Japan at 100m deep. In Taiwan, it is trawled at 400 to 500m depth off the Gueishan Island. This species is a new record from the Taiwan waters.

#### Records

*Claviscala subulae* Nakayama 2000: figs 17–18; Nakayama 2003: p1. 9, figs 19–22.

### Epitonium (Epitonium) okezoko

(Habe 1961)

#### Description

White, thin, pyramidal, teloconch rounded, suture perforated, with 14 thin erected costae which hooked at the shoulder, interspaces sculptured with thin spiral striae (Fig. 1b). Umbilicus opened. Shell height 30–40mm.

#### Distribution

This species occurs off Ashizuri Cape, Tosa Bay, Japan. In Taiwan, it is dredged at 200 to 300m depth of Tong-kang waters, the Peng-hu Trench and the Gueishan Island. This species is a new record from the Taiwan waters.

#### Records

*Cycloscala okezoko* Habe 1961: pl. 14, fig. 31; Higo et al. 1999 and Higo et al. 2001: G1893.

Epitonium (Epitonium) okezoko Okutani 2000: pl. 164, fig. 61; Nakayama 2003: pl. 11, figs 4–6.

### Epitonium (Epitonium) sororastra

Kilburn 1985

#### Description

White, small, with 7–9 costae that are thin, erect and continuous (Fig. 2a). The suture and umbilicus are perforated. The spaces between the costae are smooth, and the aperture is oval. Shell height 8–11mm.

#### Distribution

This species occurs off Sri Lanka. In Taiwan, only some dead specimens were collected on the beach of Lu-tao Island. This species is a new record from the East Asian region.

#### Records

Epitonium (Epitonium) sororastra Kilburn 1985: fig. 66; Weil et al. 1999: fig. 137.

### Epitonium (Hirtoscala) fauroti

(Jousseaume 1911)

#### Description

White, tiny, axial costae strong and incurved, usually 11–12 in number, slightly elevated at the suture to form an angle, interval smooth (Fig. 2b). Umbilicus closed. Shell height about 3mm.

#### Distribution

The specimen was collected from the Red Sea. In Taiwan, one was found on the beach at Lu-tao Island in 1995. This species is a new record from the East Asian region.

#### Records

*Turbiniscala fauroti* Jousseaume 1911: pl. 6, fig. 33–36, 42; pl. 7, figs 50–52.

*Epitonium fauroti* Kaicher 1981: No. 3111.

Epitonium (Hirtoscala) fauroti Weil et al. 1999: fig. 200.

### Epitonium (Parviscala) beachportensis

(Cotton & Godfrey, 1938)

#### Description

Tiny, white, costae about 16–18 in number, that are continuous from whorl to whorl, with a peaked angle below the suture (Fig. 2c). Interspaces between the costae sculpture with fine spiral threads, without umbilicus. Shell height 5–9mm.

#### Distribution

The species has been taken off South Australia. In Taiwan, one dead specimen was collected from the beach of Lu-tao Island. This species is a new record from the East Asian region.

#### Records

Scala (Mazescala) beachportensis Cotton and Godfrey 1938: pl. 17, figs 5.

*Epitonium beachportensis* Kaicher 1981: No. 3057.

Epitonium (Parviscala) beachportense Weil et al. 1999: p.134.

### Epitonium (Parviscala) corniculum

Lee & Huang sp. n.

urn:lsid:zoobank.org:act:9E588A81-347C-4A66-98FA-8EBA716F5BA4

#### Materials

- **Type status:** Holotype. **Occurrence:** catalogNumber: NMNS-7035-001; recordedBy: Chih-Wei Huang, Yen-Chen Lee; individualID: holotype; individualCount: 1; lifeStage: adult; disposition: dry; **Taxon:** scientificName: Epitonium (Parviscala) corniculum; acceptedNameUsage: Epitonium (Parviscala) corniculum Lee & Huang; kingdom: Animalia; phylum: Mollusca; class: Gastropoda; order: Caenogastropoda; family: Epitoniidae; genus: Epitonium; subgenus: Parviscala; specificEpithet: corniculum; scientificNameAuthorship: Lee & Huang; nomenclaturalCode: ICZN; taxonRemarks: sp. nov.; **Location:** continent: Asia; waterBody: Peng-chia-yu water, Western Pacific; islandGroup: Taiwan; island: Peng-chia-yu Island; country: Taiwan; locality: Peng-chia-yu water; minimumDepthInMeters: 500m; maximumDepthInMeters: 600m; **Identification:** identifiedBy: Yen-Chen Lee; dateIdentified: 2014; identificationRemarks: sp. nov.; **Event:** samplingProtocol: Dredging; **Record Level:** language: en; institutionID: NMNS; collectionID: NMNS-7035-001; basisOfRecord: PreservedSpecimen
- **Type status:** Paratype. **Occurrence:** catalogNumber: NMNS-7035-002; recordedBy: Chih-Wei Huang, Yen-Chen Lee; individualID: paratype 1; individualCount: 1; lifeStage: adult; disposition: dry; **Taxon:** scientificName: Epitonium (Parviscala) corniculum; acceptedNameUsage: Epitonium (Parviscala) corniculum Lee & Huang; kingdom: Animalia; phylum: Mollusca; class: Gastropoda; order: Caenogastropoda; family: Epitoniidae; genus: Epitonium; subgenus: Parviscala; specificEpithet: corniculum; scientificNameAuthorship: Lee & Huang; nomenclaturalCode: ICZN; taxonRemarks: sp. nov.; **Location:** continent: Asia; waterBody: Peng-chia-yu water, Western Pacific; islandGroup: Taiwan; island: Peng-chia-yu Island; country: Taiwan; locality: Peng-chia-yu water; minimumDepthInMeters: 500m; maximumDepthInMeters: 600m; **Identification:** identifiedBy: Yen-Chen Lee; dateIdentified: 2014; identificationRemarks: sp. nov.; **Event:** samplingProtocol: Dredging; **Record Level:** language: en; institutionID: NMNS; collectionID: NMNS-7035-002; basisOfRecord: PreservedSpecimen
- **Type status:** Paratype. **Occurrence:** catalogNumber: NMNS-7035-003; recordedBy: Chih-Wei Huang, Yen-Chen Lee; individualID: paratype 2; individualCount: 1; lifeStage: adult; disposition: dry; **Taxon:** scientificName: Epitonium (Parviscala) corniculum; acceptedNameUsage: Epitonium (Parviscala) corniculum Lee & Huang; kingdom: Animalia; phylum: Mollusca; class: Gastropoda; order: Caenogastropoda; family: Epitoniidae; genus: Epitonium; subgenus: Parviscala; specificEpithet: corniculum; scientificNameAuthorship: Lee & Huang; nomenclaturalCode: ICZN; taxonRemarks: sp. nov.; **Location:** continent: Asia; waterBody: Peng-chia-yu water, Western Pacific; islandGroup: Taiwan; island: Peng-chia-yu Island; country: Taiwan; locality: Peng-chia-yu water; minimumDepthInMeters: 500m; maximumDepthInMeters: 600m; **Identification:** identifiedBy: Yen-Chen Lee; dateIdentified: 2014; identificationRemarks: sp. nov.; **Event:** samplingProtocol: Dredging; **Record Level:** language: en; institutionID: NMNS; collectionID: NMNS-7035-003; basisOfRecord: PreservedSpecimen

#### Description

Shell acuminate. Fragilely thin, light weight, white color, shell width/height ratio approximate 0.38 (Fig. 4a, b, c). Spire elevated, convex, with 11–14 teleoconch whorls, protoconch missing in all type specimens. Body whorl shorter than 1/3 shell height. Surface with thin, erect axial costae, 22–34 in number on the body whorls, slightly serrated and raised to a hook halfway from the suture to the periphery. Each costae more or less connected with lower whorl’s costae on suture. With visible spiral strips between costae, the spiral strips are unclear or invisible near the suture. Whorls connected. The umbilicus is closed. Aperture ovate in shape, approximately 1/5 of shell height. Tortuously patulous at the terminal end of the columellar. The round operculum is brownish black. Shell height 20–25mm.

##### Measurement and type depository

Holotype: SL: 25.7mm, SW: 8.3mm; APL: 4.9mm, APW: 4.9mm; NMNS-7035-001, National Museum of Natural Science, Taiwan. (Fig. 4a)

Paratype 1: SL: 25.2mm, SW: 8.4mm; APL: 3.9mm, APW: 4.4mm; NMNS-7035-002, National Museum of Natural Science, Taiwan. (Fig. 4b)

Paratype 2 (earlier whorls lost): SL: 15.6mm, SW: 5.9mm; APL: 2.8mm, APW: 3.3mm; NMNS-7035-003, National Museum of Natural Science, Taiwan. (Fig. 4c)

#### Etymology

Latin means “hornlike”.

#### Distribution

Type locality: Dredged from Peng-chia-yu water at the depth of 500–600m.

#### Taxon discussion

This species is similar to the North Atlantic Ocean abyssal species *E. babylonium* (Fig. 4e, f) in morphology, but it can be distinguished by its gradually wider and longer shell with similar whorls of the present species. Epitonium (Parviscala) duocamurum (Fig. 4d) is another analogue, it differs in having more costae at the same whorl. *Eptonium sakuraii* Habe, 1962 is another analogue. The new species is dull, but *E. sakuraii* has a lustrous surface and, unlike the new species, has shoulder hooks away from the suture. The new species has shoulder hooks just under the suture, and it has fewer axial costae (22–34 in persent new species; 30–37 in *E. sakuraii*). The ratio of shell wide and shell height of *E. sakuraii* (NSMT-Mo 70316a possible paratype) is 0.3938, in holotype of *Viciniscala ootanii* Azuma, 1962 (synonym of *E. sakuraii*) it is 0.4060. The ratio of shell wide and shell height in the new species’ holotype, paratype 1, paratype 2 are 0.3185, 0.3250, 0.3252, respectively. In other words, the new species is more slender than *E. sakuraii*.

#### Records

*Epitonium abyssicola* (non Schepman 1909) Lee 2001a: fig. 17.

### Epitonium (Parviscala) pallidizonatum

(Masahito, Kuroda & Habe in Kuroda, Habe & Oyama, 1971)

#### Description

White, small, elongated, costae of approximately 23 in number at the last whorl, with sharply spine at the shoulder, clearly spiral cords between costae, without umbilicus (Fig. 1c). Shell height 22.2mm.

#### Distribution

Occurs in the Pacific coast off Sagami Bay to Kii Peninsula at 100m deep. In Taiwan it was dredged at about 500m depth of NE Taiwan waters. This species is a new record from the Taiwan waters.

#### Records

*Cinctiscala pallidizonatum* Masahito, Kuroda & Habe in Kuroda et al. 1971: pl. 63, figs 21.

Epitonium (Parviscala) pallidizonatum Weil et al. 1999: fig. 384; Okutani 2000: pl. 167, fig. 89; Nakayama 2003: pl. 13, figs 22–24.

### Epitonium (Parviscala) tenuipicturatum

Nakayama 2000

#### Description

White, small, elongated, suture deep, costae of approximately 28–42 in number at the last whorl, thin, reflexed, with angulate at the suture, clearly spiral cords between costae, without umbilicus (Fig. 1d). Shell height about 15–20mm.

#### Distribution

It is found from Boso Peninsula to Kii Peninsula, Japan. It was dredged at about 100m depth of NE Taiwan waters. This species is a new record from the Taiwan waters.

#### Records

Epitonium (Parviscala) tenuipicturatum Nakayama 2000: figs 19–20; Nakayama 2003: pl. 13, figs 25–29.

### Epitonium (Parviscala) yamakawai

(Yokoyama 1922)

#### Description

Tiny, white, suture deep, surface with about 13–14 incurved costae that are slightly winged at the shoulder, space between costae with clearly spiral cords, umbilicus closed (Fig. 1e). Shell height about 3–5mm.

#### Distribution

It is found from Sagami Bay to Tosa Bat, Japan. In Taiwan, it was collected from the beach of Ho-Mei, Taipei County. This species is a new record from the Taiwan waters.

#### Records

*Scalaria yamakawai* Yokoyama 1922: pl 4, fig. 17.

Epitonium (Cinctiscala) yamakawai Oyama 1973: pl. 6, fig. 18.

*Cinctiscala yamakawai* Higo et al. 1999: G1919.

Epitonium (Parviscala) yamakawai Okutani 2000: pl. 167, fig. 93; Nakayama 2003: pl. 14, figs 1–2.

### Epitonium (Sodaliscala) tryoni

(de Boury, 1913)

#### Description

Small, white, glossy, with 15–20 thin erect costae that are not peaked (Fig. 3a). With spiral cords between the costae. The whorls are convex and the suture is deep, but closed. The umbilicus is closed. The aperture is oval. Shell height is 3mm.

#### Distribution

Ranging from Iran to Pakistan to the Maldives. In Taiwan, only one dead specimen was found on the beach of Lu-tao Island. This species is a new record from the Indo-Pacific region.

#### Records

*Scala tryoni* de de Boury 1913: p.108.

*Epitonium tryoni* Kaicher 1981: No. 2379.

### Limiscala maraisi

(Kilburn 1985)

#### Description

Shell white, with 14–16 incurved low costae, which are blade-like under the suture (Fig. 3b). Has fine, closed spiral threads between costae. Umbilicus open but narrow. Shell height about 10mm. Similar to *Surrepifungium patamakanthini* A. Gittenberger & E. Gittenberger, 2005. However, the present species has thicker shell, is smaller in size, and the costae do not form a coronation.

#### Distribution

This species was original found in Transkei, South Africa. In Taiwan, it was collected on the beach of the Lu-tao Island. This species is a new record from the Indo-Pacific region.

#### Records

Epitonium (Limiscala) maraisi Kilburn 1985: fig 124; Weil et al. 1999: fig. 155.

### Plastiscala morchi

(Angas 1871)

#### Description

White, acuminate and elongated, with rough ribs on the first several whorls, surface sculptured with rough spiral cords which pass through the ribs, aperture subcircular, umbilicus closed (Fig. 1f). Shell height 10–50mm.

#### Distribution

This species ranges from New South Wales, Australia to Amami O-shima, Ryukyu, Japan. In Taiwan, it is trawled at the depth of 100 to 200m of northeastern Taiwan waters. This species is a new record from the Taiwan waters.

#### Records

Scala (Cirsotrema) morchi Angas 1871: pl. 1, fig. 7.

*Cirsotrema morchi* Kaicher 1981: No. 3106, 3046 (form *bentha* Iredale), 3130 (form *profundior* Iredale).

*Plastiscala morchi* Iredale 1936: fig. 21; Okutani 2000: pl. 160, fig. 16; Nakayama 2003: pl. 3, fig. 8.

*Plastiscala morchi bentha* Iredale 1936: fig. 22.

*Plastiscala morchi profundior* Iredale 1936: fig. 23.

### Surrepifungium ingridae

(A. Gittenberger & Goud 2000)

#### Description

Shell white, thin and fragile, spire pyramidal elevated, surface sculptured with densely lamella, which are extended at the suture (Fig. 2d). Interspaces between lamella are sculptured with fine spiral striae. The body whorl is large; almost half the height of the shell. Umbilicus narrow but distinctly perforated, partly covered by the incurved columellar. Shell height 25–30mm.

#### Distribution

This species ranges from Australia, Queensland though Indonesia. It is new records for Taiwan and the East Asian region. In 1999, several specimens were trawled at Taiwan Strait at depths of 20–50m. This species is a new record from the East Asian region.

#### Records

*Epitonium ingridae* Gittenberger et al. 2000: Figs 7–8, 23–24, 27, 30.

## Checklists

### Checklist of Epitoniidae in Taiwan

#### Acrilla acuminata

(G. B. Sowerby II 1844)

#### Alora annulata

(Kuroda & Ito 1961)

#### Amaea (Amaea) foulisi

Kilburn 1985

#### Amaea (Amaea) hedleyi

(de Boury 1912)

#### Amaea (Amaea) magnifica

(G. B. Sowerby II 1844)

#### Amaea (Amaea) ogaitoi

Masahito & Habe 1975

#### Amaea (Amaea) secunda

Kuroda & Ito 1961

#### Amaea (Amaea) thielei

(de Boury 1912)

#### Amaea (Clathroscala) cerea

(Masahito, Kuroda & Habe in Kuroda, Habe & Oyama 1971)

#### Amaea (Filiscala) reticulata

(Lee & Wu 1998)

#### Amaea (Filiscala) rubigosola

Lee 2001

#### Amaea (Scalina) flammea

Lee 2001

#### Amaea (Scalina) gazeoides

Kuroda & Habe in Habe 1961

#### Amaea (Scalina) oyasionensis

(Ozaki 1958)

#### Amaea (Scalina) sericogazea

(Masahito, Kuroda & Habe in Kuroda, Habe & Oyama 1971)

#### Amaea (Scalina) splendida

(de Boury 1913)

#### Amaea nebulodermata

(Azuma 1972)

#### Cirsotrema (Cirsotrema) bonum

(Melvill 1906)

#### Cirsotrema (Cirsotrema) cloveri

Brown 2002

#### Cirsotrema (Cirsotrema) varicosum

Lamarck 1822)

#### Cirsotrema (Elegantiscala) edgari

(de Boury 1912)

#### Cirsotrema (Elegantiscala) rugosum

Kuroda & Ito 1961

#### Claviscala subulae

Nakayama 2000

#### Claviscala terebralioides

(Kilburn 1975)

#### Cycloscala crenulata

(Pease 1867)

#### Cycloscala hyalina

(G. B. Sowerby II 1844)

#### Cylindriscala nitida

(Kuroda & Ito 1961)

#### Cylindriscala solar

(Nakayama 1995)

#### Eglisia lanceolata

Reeve 1849

#### Eglisia tricarinata

A. Adams & Reeve 1850

#### Epidendrium aureum

Gittenberger, A. & Gittenberger, E., 2005

#### Epitonium (Depressiscala) aureomaculatum

(Masahito & Habe 1973)

#### Epitonium (Depressiscala) umbilicatum

(Pease 1869)

#### Epitonium (Epitonium) alatum

(G. B. Sowerby II 1844)

#### Epitonium (Epitonium) laxatoides

Kuroda in Nakayama 1995

#### Epitonium (Epitonium) liliputanum

(A. Adams 1861)

#### Epitonium (Epitonium) okezoko

(Habe 1961)

#### Epitonium (Epitonium) pallasii pallasii

(Kiener 1838)

#### Epitonium (Epitonium) pallasii neglectum

(A. Adams & Reeve 1850)

#### Epitonium (Epitonium) parspeciosum

(Iredale 1929)

##### Notes

new record of Taiwan

#### Epitonium (Epitonium) profundum

Nakayama 2000

#### Epitonium (Epitonium) scalare

(Linné 1758)

#### Epitonium (Epitonium) sororastra

Kilburn 1985

#### Epitonium (Epitonium) syoichiroi

Masahito & Habe 1976

#### Epitonium (Epitonium) tokyoense

(Kuroda 1930)

#### Epitonium (Glabriscala) glabratum

(Hinds 1843)

##### Notes

new record of East Asian region

#### Epitonium (Glabriscala) hayashii

(Habe 1961)

#### Epitonium (Glabriscala) stigmaticum

(Pilsbry 1911)

#### Epitonium (Hirtoscala) fauroti

(Jousseaume 1911)

#### Epitonium (Hirtoscala) ferussacii

(Audouin 1826)

#### Epitonium (Hirtoscala) pyramidale

(G. B. Sowerby II 1844)

#### Epitonium (Hirtoscala) tenuicostatum

(G. B. Sowerby II 1844)

##### Notes

new record of East Asian region

#### Epitonium (Hyaloscala) calideum

(Melvill & Standem 1903)

#### Epitonium (Hyaloscala) jukesianum

(Forbes, 1852)

#### Epitonium (Hyaloscala) kraussi

(Nyst 1871)

#### Epitonium (Laeviscala) fucatum

(Pease 1861)

#### Epitonium (Laeviscala) gracile

(G. B. Sowerby II 1844)

#### Epitonium (Lamelliscala) abyssicola

(Schepman 1909)

#### Epitonium (Lamelliscala) aculeatum

(G. B. Sowerby II 1844)

#### Epitonium (Lamelliscala) climacotum

(Kilburn 1985)

#### Epitonium (Lamelliscala) coretum

(Iredale 1936)

#### Epitonium (Lamelliscala) gravieri

(Jousseaume 1911)

#### Epitonium (Lamelliscala) philippinarum

(G. B. Sowerby II 1844)

#### Epitonium (Mazescala) bellicosum

Hedley 1907

#### Epitonium (Nitidiscala) angustum

(Dunker, 1861)

#### Epitonium (Nitidiscala) synekhes

Kilburn 1985

#### Epitonium (Papyriscala) catanuense

(G. B. Sowerby II 1844)

#### Epitonium (Papyriscala) imperiale

(G. B. Sowerby II 1844)

#### Epitonium (Papyriscala) robillardi

(Sowerby 1894)

#### Epitonium (Papyriscala) tenuiliratum

(G. B. Sowerby II 1844)

#### Epitonium (Parviscala) beachportense

(Cotton & Godfrey, 1938)

#### Epitonium (Parviscala) chinglinae

Lee & Wu 1998

#### Epitonium (Parviscala) corniculum

sp. nov.

#### Epitonium (Parviscala) duocamurum

Lee 2001

#### Epitonium (Parviscala) eximium

(A. Adam & Reeve 1850)

##### Notes

new record of East Asian region

#### Epitonium (Parviscala) gradile

(Jousseume 1911)

#### Epitonium (Parviscala) grossicingulatum

de Boury, 1913

#### Epitonium (Parviscala) harpago

Kilburn 1985

#### Epitonium (Parviscala) obliquum

(G. B. Sowerby II 1844)

#### Epitonium (Parviscala) pallidizonatum

(Masahito, Kuroda & Habe in Kuroda, Habe & Oyama, 1971)

##### Notes

new record of Taiwan

#### Epitonium (Parviscala) paumotense

(Pease, 1867)

#### Epitonium (Parviscala) repandum

Kilburn 1985

#### Epitonium (Parviscala) tenuipicturatum

Nakayama 2000

#### Epitonium (Parviscala) townsendi

(Melvill & Standen 1903)

#### Epitonium (Parviscala) yamakawai

(Yokoyama 1922)

#### Epitonium (Sodaliscala) immaculata

(G. B. Sowerby II 1844)

##### Notes

new record of Taiwan

#### Epitonium (Sodaliscala) mindoroense

(G. B. Sowerby II 1844)

#### Epitonium (Sodaliscala) multicostatum

(G. B. Sowerby II 1844)

##### Notes

new record of Taiwan

#### Epitonium (Sodaliscala) pasiphaes

(Melvill 1912)

#### Epitonium (Sodaliscala) symmetricum

(Pease 1867)

#### Epitonium (Sodaliscala) tryoni

(de Boury, 1913)

#### Epitonium (Sodaliscala) zatrephes

(Melvill 1910)

#### Epitonium (Strephoscala) taiwanica

Lee & Wu 1998

#### Epitonium tosaensis

(Azuma 1962)

#### Filiscala raricosta

(Lamarck, 1804)

#### Fragilopalia lotus

(Masahito & Habe 1975)

#### Globiscala bullata

(G. B. Sowerby II 1844)

#### Graciliscala rimbogai

Masahito & Habe 1976

#### Gyroscala (Circuloscala) iwaotakii

(Azuma 1961)

#### Gyroscala (Circuloscala) watanabei

Nakayama 2000

#### Gyroscala (Pomiscala) lamellosa

(Lamarck 1822)

#### Librariscala parvonatrix

(Kilburn 1985)

#### Limiscala crypticocorona

(Kilburn 1985)

#### Limiscala irregulare

(G. B. Sowerby II 1844)

#### Limiscala lyra

(G. B. Sowerby II 1844)

#### Limiscala maraisi

(Kilburn 1985)

#### Limiscala virgo

(Masahito & Habe 1976)

#### Narvaliscala percancellata

(Nakayama 2000)

#### Opalia bardeyi

(Jousseaume 1911)

##### Notes

new record of Indo–Pacific region

#### Opalia bicarinata

(G. B. Sowerby II 1844)

## Discussion

Most species of this family are white or brown in color and have circular apertures. These delicate shells are generally pyramidal or drop-shaped with many axial costae. The paucispiral horny opercula are black or translucent yellow. They are distributed from tidal to great depths in sandy areas in most seas and found on corals or sea anemones, which feed on them (Nakayama 2000, Gittenberger et al. 2000, Gittenberger and Gittenberger 2005). Most epitoniids are protandrous (Robertson 1981), but the ecology of deep water species has not been documented. They have a ptenoglossan radula with a broad expanse of small, sickle-like teeth (Azuma 1971).

Most Taiwanese wentletraps are rare and hard to obtain, even when dead, because of their tiny size or deep water habitat. These small species are difficult to identify because of their overall similarity and few references. The radula is not informative for generic classification, although protoconch morphology has been used to distinguish the genera (Kilburn 1985). The key characters used to identify epitoniids are costae characters—whether the shell has a basal ridge or is shaped like a conch (Kilburn 1985, Nakayama 2000). The costal characters are more important than others. We focus on the shape of the costae, which includes their thickness, breadth and coiling, and whether the costae are erect or appear serrated, but not the numbers wach specimen has. The microscopically striated sculpture of the spaces between costae is also an important character.

Kuroda (1941) listed 6 unidentified species. Three of them have Japanese name. They are キヌイトカケ, キヌメセキモリ and チリメンイトカケ. Their scientific name are *Limiscala irregular*, *Limiscala lyra* and *Amaea immaculata* through the check of Japanese name on the list of Higo and Goto (1993).

Before our investigation, 101 epitoniids species were recorded in Taiwan (Kuroda 1941, Hayasaka 1944, Lan 1976, Lan 1999, Shih and Wan 1982, Chan et al. 1982, Chang 1982, Chong 1993, Lai 1998, Su 1999, Jeng et al. 1994, Jeng et al. 1996, Jeng et al. 1997, Jeng et al. 1998, Jeng et al. 2000, Lee and Wu 1998, Lee 2001a, Lee 2001b, Lee 2003, Chen and Lee 2007, Lee and Wu 2012). Based on our investigations of the coasts and fishing ports of Taiwan and illustrations by previous investigators, a total of 114 species belonging to 23 genera of Epitoniidae are reported in Taiwan. Of these, 12 are new records in Taiwan, including 4 new that are new to the East Asian region, 2 that are new to the Indo-Pacific region and 1 new species.

## Figures and Tables

**Figure 1a.**
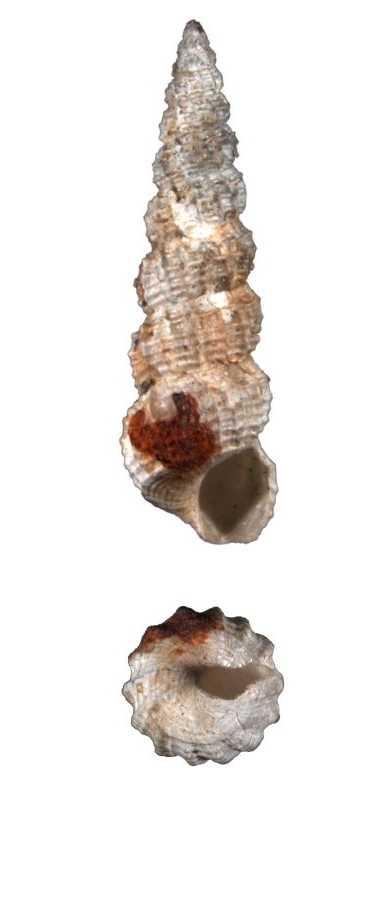
*Claviscala subulae* Nakayama 2000, 11.7×3.5mm

**Figure 1b.**
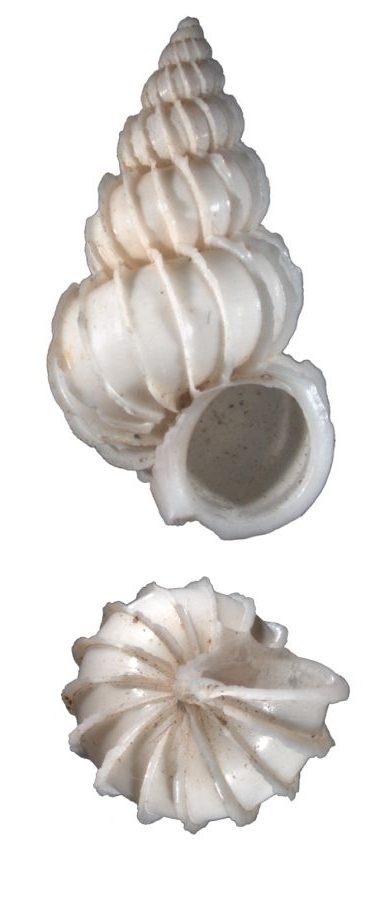
Epitonium (Epitonium) okezoko (Habe 1961), 21.6×11.9mm

**Figure 1c.**
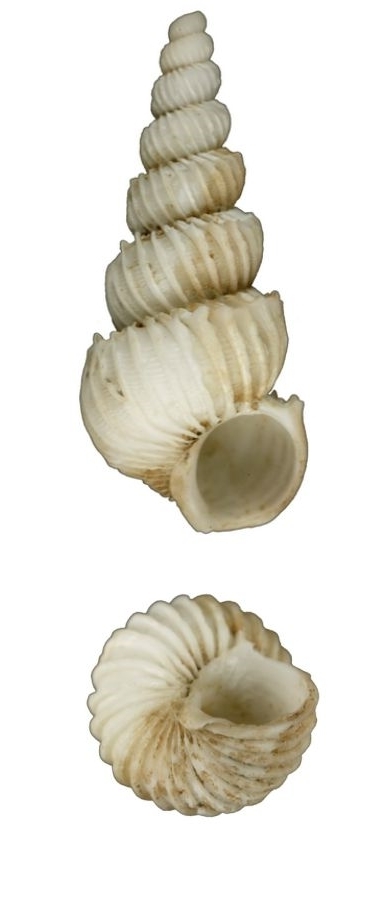
Epitonium (Parviscala) cf. pallidizonatum (Masahito, Kuroda & Habe in Kuroda, Habe & Oyama, 1971), 22.2×9.7mm

**Figure 1d.**
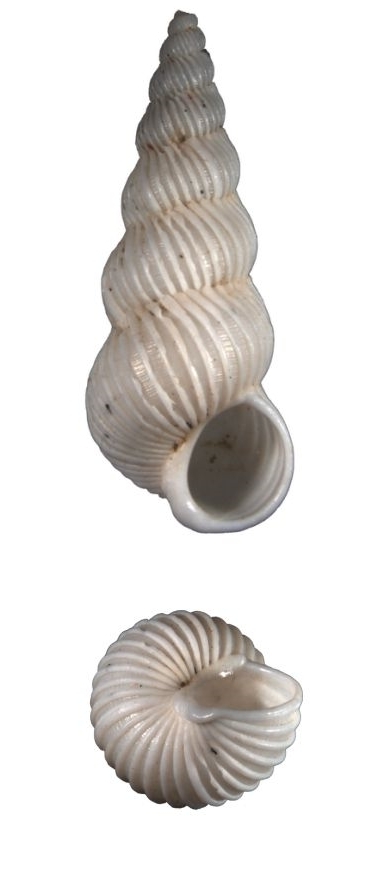
Epitonium (Parviscala) tenuipicturatum Nakayama 2000, 13.6×5.4mm

**Figure 1e.**
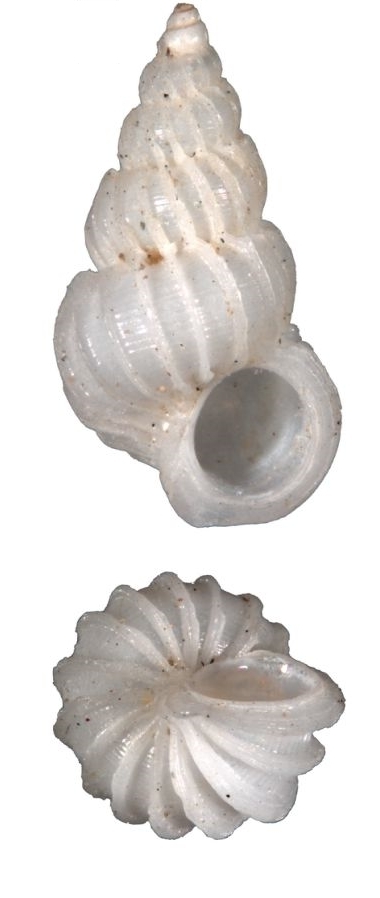
Epitonium (Parviscala) yamakawai (Yokoyama 1922), 3.3×1.8mm

**Figure 1f.**
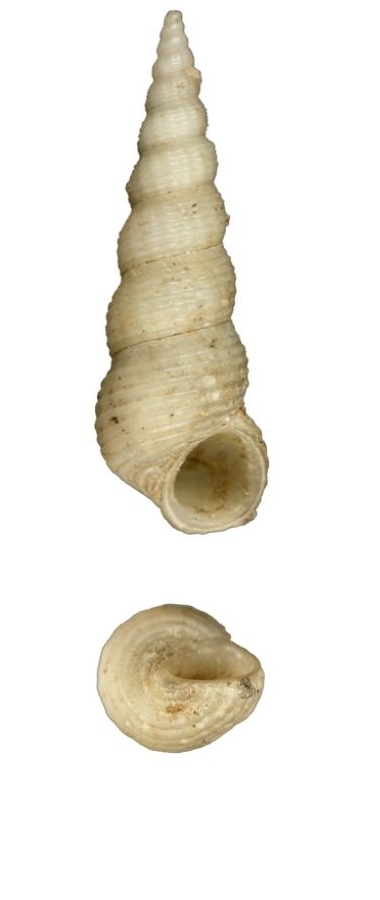
*Plastiscala morchi* (Angas 1871), 12.5×4.1mm

**Figure 2a.**
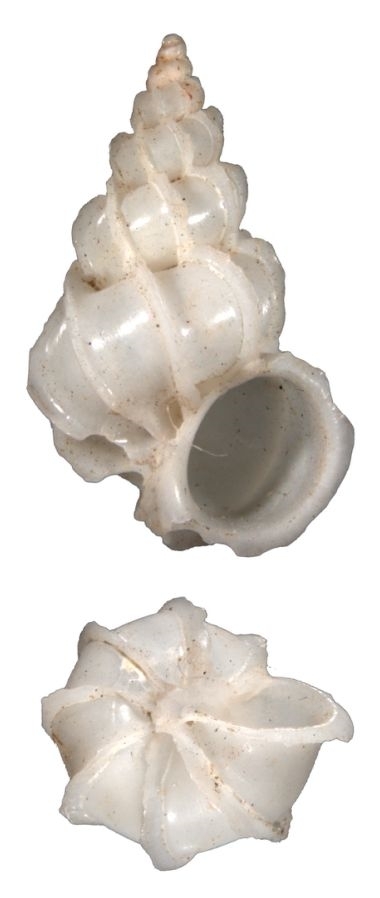
Epitonium (Epitonium) sororastra Kilburn 1985, 7.8×5mm

**Figure 2b.**
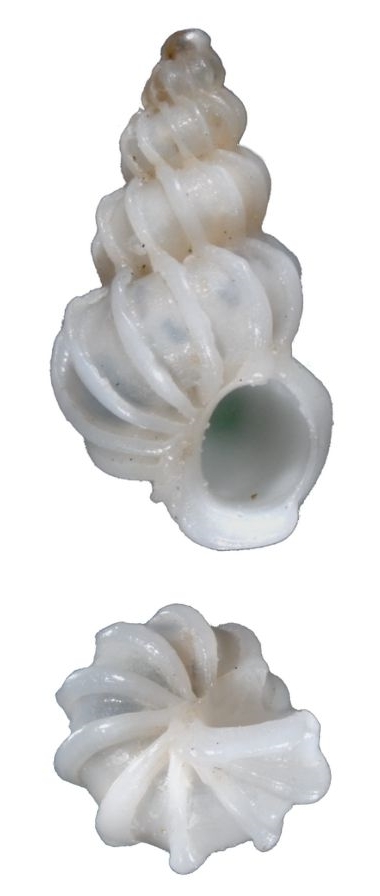
Epitonium (Hirtoscala) fauroti (Jousseaume 1911), 2.8×1.5mm

**Figure 2c.**
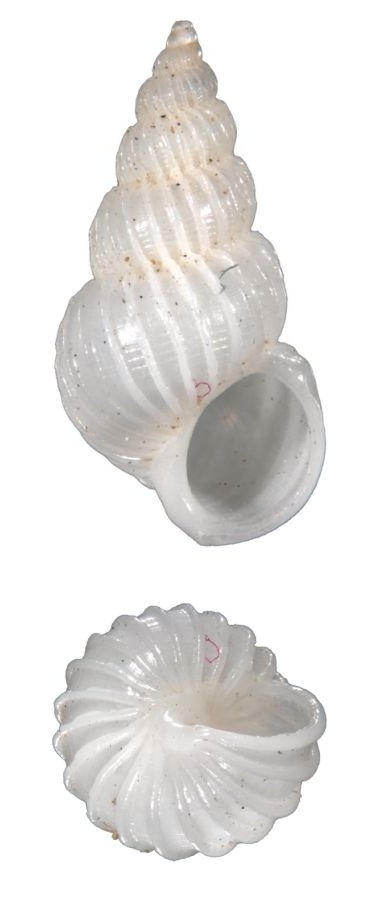
Epitonium (Parviscala) beachportensis (Cotton & Godfrey, 1938), 5.2×2.6mm

**Figure 2d.**
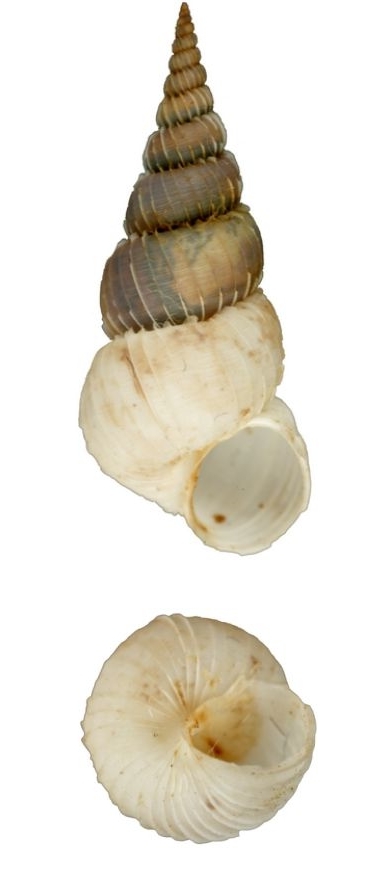
*Surrepifungium ingridae* (A. Gittenberger & Goud 2000), 23.9×10.1mm

**Figure 3a.**
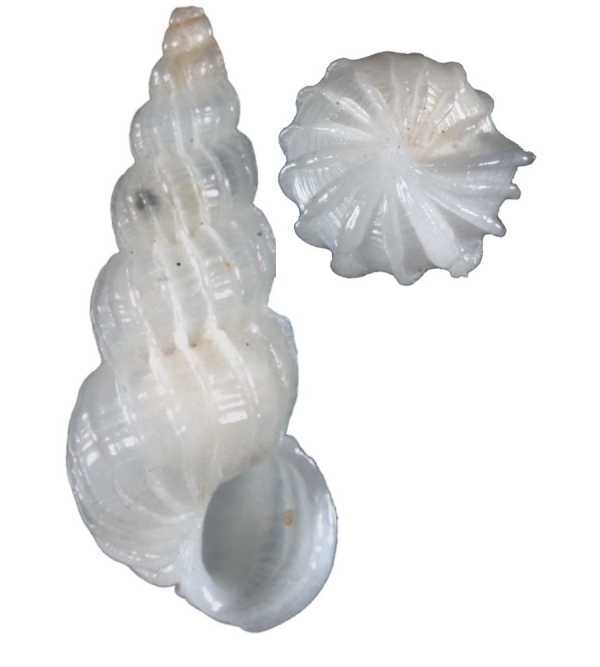
Epitonium (Sodaliscala) tryoni (de Boury, 1913), 3.1×1.3mm

**Figure 3b.**
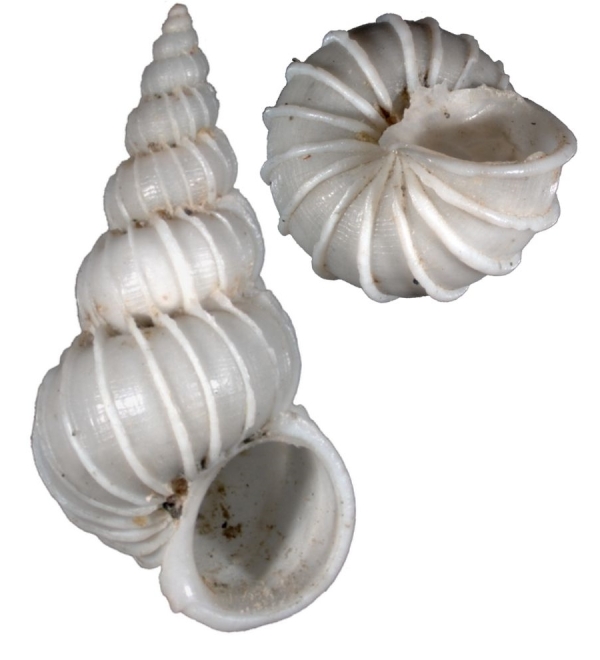
*Limiscala maraisi* (Kilburn 1985), 11.2×5.8mm

**Figure 4a.**
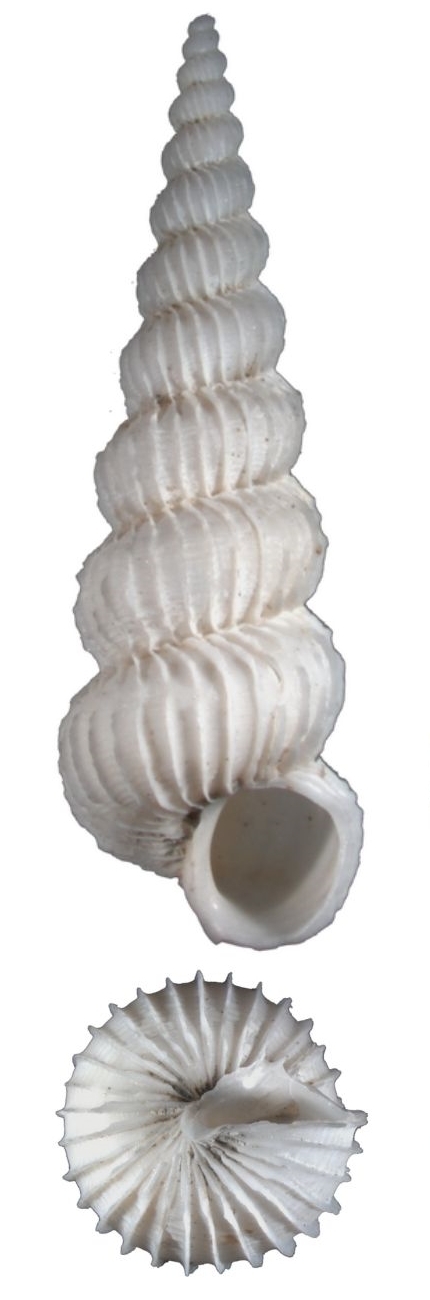
E. (P.) corniculum, holotype, 25.7×8.3mm

**Figure 4b.**
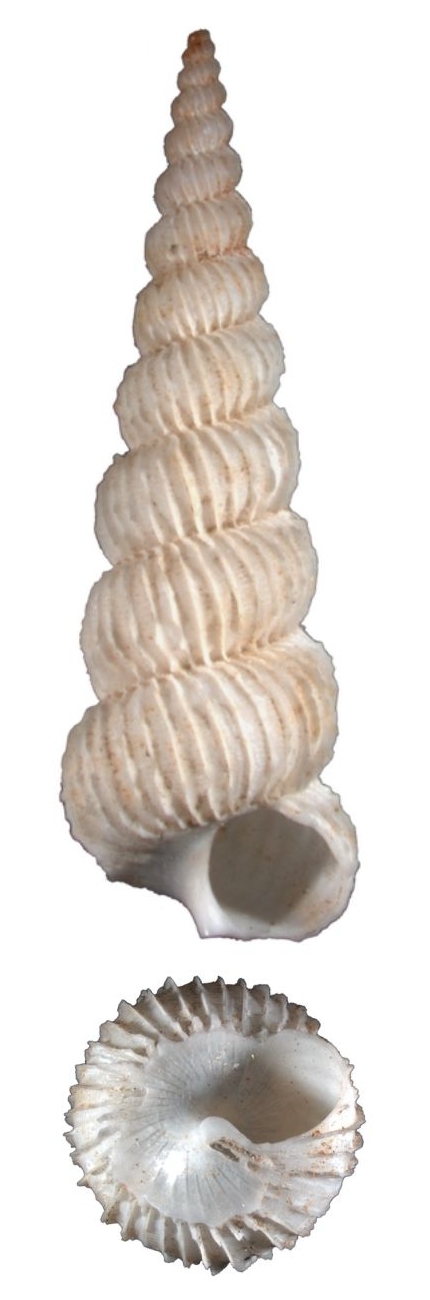
E. (P.) corniculum, paratype 1, 25.2×8.4mm

**Figure 4c.**
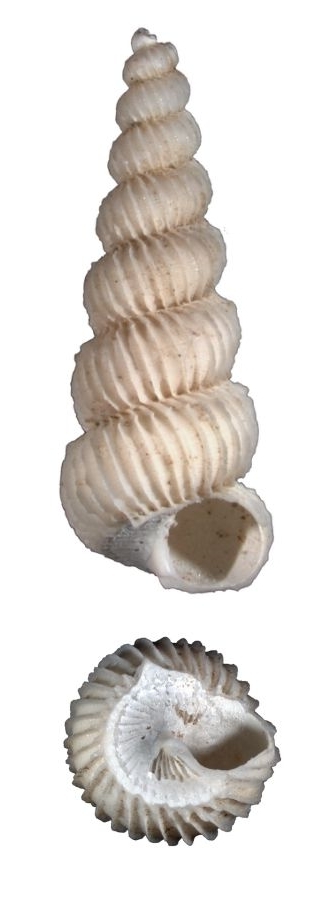
E. (P.) corniculum, paratype 2, 15.6×5.9mm (earlier whorls lost)

**Figure 4d.**
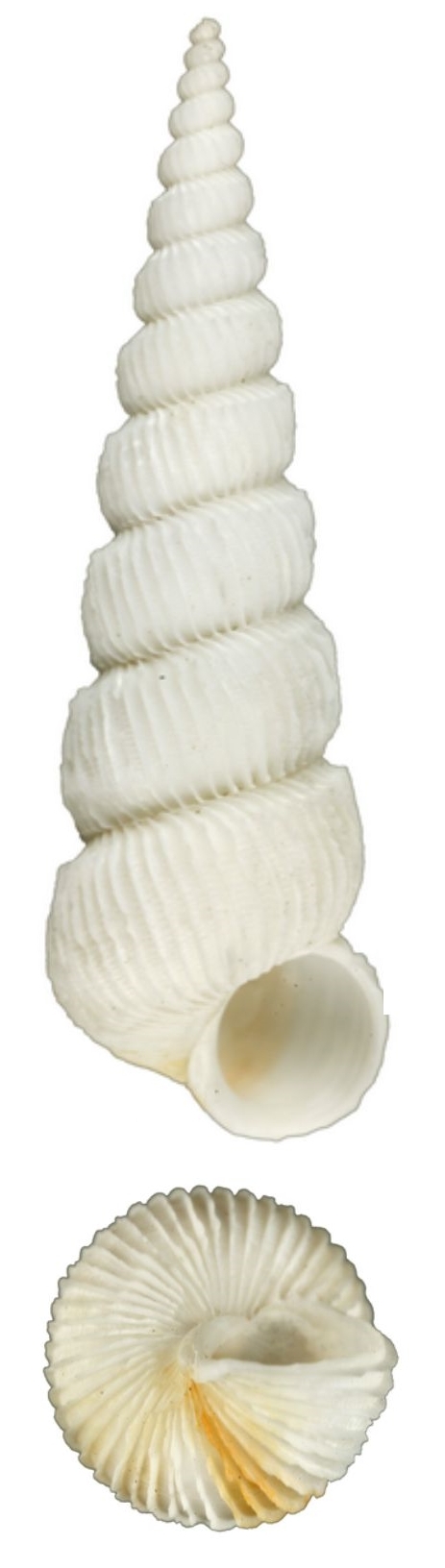
Epitonium (Parviscala) duocamurum Lee 2001, 33×11.8mm

**Figure 4e.**
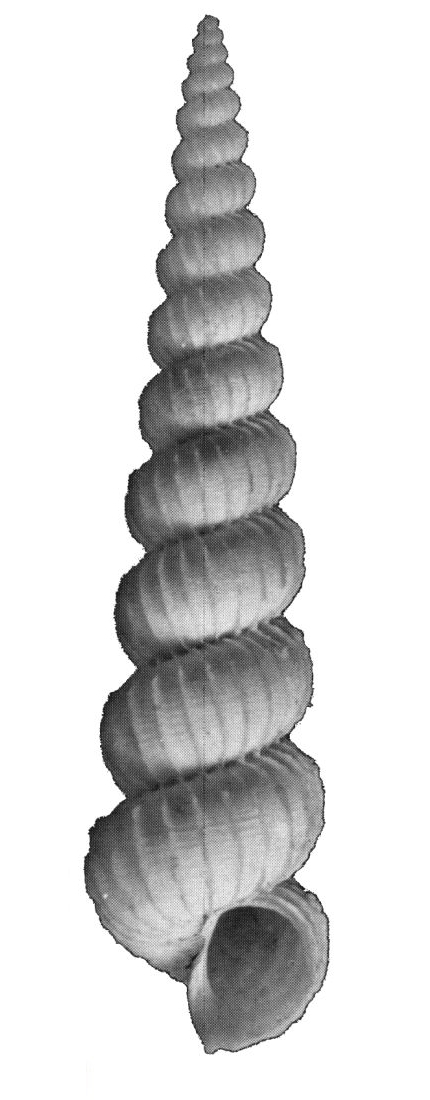
E. (Asperiscala) babylonium (Dall 1889), 28.5×6.5mm (holotype)

**Figure 4f.**
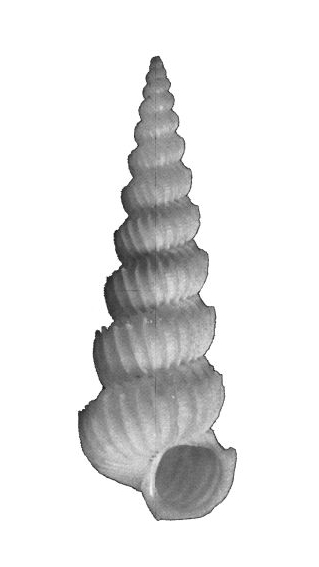
E. (Asperiscala) babylonium (Dall 1889), 12.8×4.5mm (Clench & Turner 1952, p315, figs 1–2)
